# The Birmingham General Dispensary

**Published:** 1889-11-23

**Authors:** 


### The Hospital Workshop.

### No. XVI.—THE BIRMINGHAM GENERAL DISPENSARY.

(BY OUR SPECIAL COMMISSIONER.)

During the ninety-eight years of its existence this charity has carried on a work of much importance among the poor of Birmingham. The main building is in Union-street, with branches at Highgate, Nechells, and Ladywood. Patients are seen at nine in the morning by the resident medical officer, and also by a specially appointed surgeon, a general practitioner of the town, who attends several hours daily for the purpose. Attached to the staff are two consultants, a surgeon, and a physician, each of whom receives an annual payment of one hundred and fifty guineas. This somewhat unusual feature may be regarded as a sign of the times, for people appear to be finding out that honorary services are not invariably the outcome of disinterested motives. The main work of the institution, however, consists in visiting patients at their own homes, and is done by the resident medical officers, who divide the whole of Birmingham between them into convenient districts.

Admission is by subscribers' tickets, the number thus admitted during 1888 being 23,707. Of teeth cases and accidents, which are treated without tickets, there were 3,337 during the same year, distributed among the various branches as follows:

| | Teeth. | Accidents. |
|---|---|---|
| Union-street | 825 | 24 |
| Highgate | 1,652 | 66 |
| Nechells | 573 | 68 |
| Ladywood | 125 | 4 |

The sum total of applications attended to in the year mentioned is thus brought up to 27,044.

Each patient is entitled to one month's treatment and medicine, and should the case require further care at the end of that time, another ticket must be obtained. These "supernumerary" tickets, as they are called, can be purchased either by the outside public or by the patients themselves, a fact which is hardly consistent with one's preconceived notions of charity. The society's report states the average cost of each patient to be 4s. 3d. Anyone whose time of attendance is finished can go to the secretary and purchase the right of another month's treatment for four shillings—in other words, he practically pays the cost of what he receives. It is not altogether surprising, therefore, that many of the people found in these dispensaries claim as a right what they should rather accept as a bounty. Another weak point of the system lies in the fact that no investigation is made into the means of applicants. The committee seek to throw that responsibility upon subscribers, with the inevitable result that enquiry is, except in the rarest instances, altogether shelved.

In making these remarks it should be clearly understood they are not levelled at any particular institution, but against the system generally. A further defect in almost all the large charitable dispensaries is the miserably small salaries paid to the resident medical officers. In Birmingham these gentlemen receive rather more than the average pay of a house surgeon, namely, £100, but they are infinitely worse off, for the cost of board has to be deducted from that sum. There seems to be a lack of generosity about the proceeding, when it is considered that the brunt of the work falls on these officials. Their districts are large, and have not infrequently to be visited from one end to the other in the course of a single day. It is true that an allowance is made to each resident for cab fares, but being young men with restricted means they naturally enough do all their visiting on foot. If one might be allowed to make the suggestion, it would be advisable in the interests both of subscribers and of patients to increase the salaries of the officers in question, and, at the same time, to make cab hire compulsory. Such a course would not embarrass the charity, for it is a wealthy one, with a yearly income of £7,435, in part derived from endowments.

Many of the cases that come under treatment have been rejected by the hospitals as chronic or hopeless, while others have no more money to pay their own doctors, and a certain number, again, belong to the fairly extensive class that prefers getting advice and physic for nothing. A large proportion of the patients suffer from phthisis. They cannot obtain admission to the hospitals and would rather, as a rule, end their days among the surroundings of the most squalid poverty than in the workhouse infirmary, which presents their only other alternative. Your commissioner, with a tolerably wide experience, does not remember ever having seen such a collection of phthisical patients outside a hospital for consumption. In the course of his enquiries, he visited one house where two cousins, a girl of eighteen and a boy of seven, were dying in the same room from that complaint; and the half-dozen adjoining streets furnished an equal number of tubercular cases in more or less advanced stages.

To some extent the prevalence of this disease is traceable to peculiar trades, and direct heredity is, of course, an extremely common predisposing factor. Now that we are arriving at more definite conclusions as to the origin of tubercle in germ-contagion, preventive medicine may one day hope to make headway against its ravages. Among the chief measures of prevention are the destruction of animals affected with tuberculosis, the isolation of patients, and the disinfection of sputa. Your commissioner met with one case where a man attempted suicide by drinking the whole of his cough mixture, a tincture of codeia, which is a powerful alkaloid of opium. The poor fellow was wretchedly poor, and had been unable to earn anything for years, during which time his wife had toiled early and late to keep him out of the workhouse. This state of affairs preyed so much on the unfortunate man's mind that, in a fit of melancholia, he attempted to take his life in the manner mentioned.

The good work of the institution is greatly aided by the district visiting nurses, who are always ready to undertake dressings, the taking of temperatures, and other practical treatment. Then there are various relief funds attached to the charity, by which the medical officers are able, at discretion, to afford substantial help in the shape of beef, mutton, brandy, and other articles. A complete system of relief is thus approached, and some reformers look to an extension of the dispensary plan as offering a possible solution of the vexed question of out-patient treatment, and also of the proper share of general practitioners in charitable appointments, from which they are now almost entirely excluded. Committees, however, should avoid giving these posts to those who are already on the staff of other hospitals. Pluralities of this kind are as undesirable in this as in most other cases.

The dispensing department is well provided with a supply of good and pure drugs, and everything is arranged with a view to the rapid disposal of prescriptions. An idea of the amount of work entailed may be gathered from the fact that the drug bill for 1888 was over £1,300 in amount.

